# Supplementary material for: OpenMM 7: Rapid development of high performance algorithms for molecular dynamics
Source: PLoS Comput Biol. 2017 Jul 26;13(7):e1005659. doi: 10.1371/journal.pcbi.1005659 (PMC5549999; doi:10.1371/journal.pcbi.1005659)
Supplement: S1 Source Code — (ZIP) [file pcbi.1005659.s002.zip › OpenMM-7.0.1-Source/docs/api-python/generated/simtk.openmm.app.charmmparameterset.CharmmParameterSet.html]

CharmmParameterSet — OpenMM 7.0.1.dev-5e86c4f documentation


# CharmmParameterSet¶

*class* `simtk.openmm.app.charmmparameterset.``CharmmParameterSet`(*\*args*, *\*\*kwargs*)¶
:   Stores a parameter set defined by CHARMM files. It stores the equivalent of
    the information found in the MASS section of the CHARMM topology file
    (TOP/RTF) and all of the information in the parameter files (PAR)

    |  |  |
    | --- | --- |
    | Parameters: | **filenames** (*List of topology, parameter, and stream files to load into the parameter set.*) – The following file type suffixes are recognized. Unrecognized file types raise a TypeError \* .rtf, .top – Residue topology file \* .par, .prm – Parameter file \* .str – Stream file \* .inp – If “par” is in the file name, it is a parameter file. If  “top” is in the file name, it is a topology file. Otherwise, raise TypeError |

    `All type lists are dictionaries whose keys are tuples (with however`

    `many elements are needed to define that type of parameter). The types`

    `that can be in any order are SORTED.`

    `- atom_types_str`

    `- atom_types_int`

    `- atom_types_tuple`

    `- bond_types`

    `- angle_types`

    `- urey_bradley_types`

    `- dihedral_types`

    `- improper_types`

    `- cmap_types`

    `- nbfix_types`

    `The dihedral types can be multiterm, so the values for each dict key is`

    `actually a list of DihedralType instances. The atom_types are dicts that`

    `match the name (str), number (int), or (name, number) tuple (tuple) to`

    `the atom type. The tuple is guaranteed to be the most robust, although`

    `when only the integer or string is available the other dictionaries are`

    `helpful`¶

    Examples

    ```
    >>> params = CharmmParameterSet('charmm22.top', 'charmm22.par', 'file.str')
    ```

    `__init__`(*\*args*, *\*\*kwargs*)¶

    Methods

    |  |  |
    | --- | --- |
    | `__init__`(\*args, \*\*kwargs) |  |
    | `condense`() | This function goes through each of the parameter type dicts and eliminates duplicate types. |
    | `loadSet`([tfile, pfile, sfiles, permissive]) | Instantiates a CharmmParameterSet from a Topology file and a Parameter |
    | `readParameterFile`(pfile[, permissive]) | Reads all of the parameters from a parameter file. |
    | `readStreamFile`(sfile) | Reads RTF and PAR sections from a stream file and dispatches the |
    | `readTopologyFile`(tfile) | Reads \_only\_ the atom type definitions from a topology file. |

    *classmethod* `loadSet`(*tfile=None*, *pfile=None*, *sfiles=[]*, *permissive=False*)¶
    :   Instantiates a CharmmParameterSet from a Topology file and a Parameter
        file (or just a Parameter file if it has all information)

        tfile : str
        :   Name of the Topology (RTF/TOP) file

        pfile : str
        :   Name of the Parameter (PAR) file

        sfiles : list of str
        :   List or tuple of stream (STR) file names.

        permissive : bool=False
        :   Accept non-bonbded parameters for undefined atom types

        |  |  |
        | --- | --- |
        | Returns: | New CharmmParameterSet populated with the parameters found in the provided files. |
        | Return type: | CharmmParameterSet |

        Notes

        The RTF file is read first (if provided), followed by the PAR file,
        followed by the list of stream files (in the order they are
        provided). Parameters in each stream file will overwrite those that
        came before (or simply append to the existing set if they are
        different)

    `readParameterFile`(*pfile*, *permissive=False*)¶
    :   Reads all of the parameters from a parameter file. Versions 36 and later
        of the CHARMM force field files have an ATOMS section defining all of
        the atom types. Older versions need to load this information from the
        RTF/TOP files.

        |  |  |
        | --- | --- |
        | Parameters: | - **pfile** (*str*) – Name of the CHARMM PARameter file to read - **permissive** (*bool*) – Accept non-bonbded parameters for undefined atom types (default:   False). |

        Notes

        The atom types must all be loaded by the end of this routine. Either
        supply a PAR file with atom definitions in them or read in a RTF/TOP
        file first. Failure to do so will result in a raised RuntimeError.

    `readTopologyFile`(*tfile*)¶
    :   Reads \_only\_ the atom type definitions from a topology file. This is
        unnecessary for versions 36 and later of the CHARMM force field.

        |  |  |
        | --- | --- |
        | Parameters: | **tfile** (*str*) – : Name of the CHARMM TOPology file to read |

        Notes

        The CHARMM TOPology file is also called a Residue Topology File

    `readStreamFile`(*sfile*)¶
    :   Reads RTF and PAR sections from a stream file and dispatches the
        sections to readTopologyFile or readParameterFile

        |  |  |
        | --- | --- |
        | Parameters: | **sfile** (*str or CharmmStreamFile*) – Stream file to parse |

    `condense`()¶
    :   This function goes through each of the parameter type dicts and
        eliminates duplicate types. After calling this function, every unique
        bond, angle, dihedral, improper, or cmap type will pair with EVERY key
        in the type mapping dictionaries that points to the equivalent type

        Example

        ```
        >>> params = CharmmParameterSet('charmm.prm').condense()
        ```

    `__delattr__`¶
    :   x.\_\_delattr\_\_(‘name’) <==> del x.name

    `__format__`()¶
    :   default object formatter

    `__getattribute__`¶
    :   x.\_\_getattribute\_\_(‘name’) <==> x.name

    `__hash__`¶

    `__reduce__`()¶
    :   helper for pickle

    `__reduce_ex__`()¶
    :   helper for pickle

    `__repr__`¶

    `__setattr__`¶
    :   x.\_\_setattr\_\_(‘name’, value) <==> x.name = value

    `__sizeof__`() → int¶
    :   size of object in memory, in bytes

    `__str__`¶

High performance molecular simulation on GPUs


### Navigation

- Application Layer
  - Loaders and Setup
  - Representation and Manipulation
  - Simulation
  - Reporting Output
  - Extras
- Library Layer

©2015, Stanford University and the Authors.
|
Powered by Sphinx 1.3.1
& Alabaster 0.7.7
|
Page source
